# Clinical profiles, outcomes and risk factors among type 2 diabetic inpatients with diabetic ketoacidosis and hyperglycemic hyperosmolar state: a hospital-based analysis over a 6-year period

**DOI:** 10.1186/s12902-020-00659-5

**Published:** 2020-12-14

**Authors:** Xiao-yan Wu, Dun-min She, Fang Wang, Gang Guo, Ran Li, Ping Fang, Ling Li, Yun Zhou, Ke-qin Zhang, Ying Xue

**Affiliations:** 1 Department of Endocrinology, Tongji Hospital of Tongji University, Tongji University School of Medicine, Shanghai, 200065 China; 2 Shanghai Hongkou District Liangcheng New Village Street Community Health Service Center, 200434, Shanghai, China; 3 Department of Endocrinology and Metabolism, Northern Jiangsu People’s Hospital, Yangzhou, 225000 China; 4 Department of Endocrinology, People’s Hospital of Shanghai Putuo District, Shanghai, 200060 China; 5 Department of Emergency, Tongji Hospital of Tongji University, Tongji University School of Medicine, Shanghai, 200065 China

**Keywords:** Type 2 diabetes mellitus, Diabetic ketoacidosis, Hyperglycemic hyperosmolar status, Blood urea nitrogen, Continuous subcutaneous insulin infusion therapy

## Abstract

**Objective:**

Diabetic ketoacidosis (DKA) and hyperosmolar hyperglycemic state (HHS) are the two most common hyperglycemic emergencies (HEs) associated with diabetes mellitus. Individuals with HEs can present with combined features of DKA and HHS. The objective of this study is to assess the clinical characteristics, therapeutic outcomes, and associated predisposing factors of type 2 diabetic patients with isolated or combined HEs in China.

**Methods:**

We performed a retrospective analysis of 158 patients with type 2 diabetes (T2DM), complicated with DKA, HHS, or DKA combined with HHS (DKA-HHS) in Shanghai Tongji Hospital, China from 2010 to 2015. Admission clinical features, therapeutic approaches and treatment outcomes of those patients were extracted and analyzed.

**Results:**

Of the 158 patients with T2DM, 65 (41.1%) patients were DKA, 74 (46.8%) were HHS, and 19 (12.0%) were DKA-HHS. The most common precipitants were infections (111, 70.3%), newly diagnosed diabetes (28,17.7%) and non-compliance to medications (9, 5.7%). DKA patients were divided into mild, moderate and severe group, based on arterial blood gas. Spearman correlation analysis revealed that C-reaction protein (CRP) was positively correlated with severity of DKA, whereas age and fasting C peptide were inversely correlated with severity of DKA (*P* < 0.05). The mortality was 10.8% (17/158) in total and 21.6% (16/74) in the HHS group, 5.9% (1/17) in DKA-HHS. Spearman correlation analysis indicated that death in patients with HHS was positively correlated to effective plasma osmolality (EPO), renal function indicators and hepatic enzymes, while inversely associated with the continuous subcutaneous insulin infusion (CSII) therapy. Logistic regression analysis suggested that elevated blood urea nitrogen (BUN) on admission was an independent predisposing factor of mortality in HHS, while CSII might be a protective factor for patients with HHS. Furthermore, the receiver-operating characteristic (ROC) curve analysis indicated that BUN had the largest area under the ROC curves for predicting death in patients with HHS.

**Conclusions:**

Our findings showed elevated CRP and decreased fasting C-peptide might serve as indicator for severe DKA. Elevated BUN might be an independent predictor of mortality in patients with HHS, whereas CSII might be a protective factor against death in HHS.

## Background

Diabetes mellitus is a globally prevalent systemic metabolic disease which is the fifth most common cause of death worldwide [1]. Diabetic ketoacidosis (DKA) and hyperosmolar hyperglycemic state (HHS), known as hyperglycemic emergencies (HEs) of diabetes, are two most common life-threatening acute complications of diabetes [2]. DKA is prone to occur in patients with Type 1 diabetes (T1DM) spontaneously [3], yet several studies suggested that both of type 1 and type 2 diabetes may be associated with DKA. Type 2 diabetic patients may account for as much as one-third of all DKA cases [3]. Previous researches on DKA were often conducted in developed countries such as Europe, America, or Japan [4–6], nevertheless so far research data of patients with HEs from mainland China were limited, especially on the overall precipitants, clinical features, treatment outcomes and risk factors.

HHS is the most dangerous complication of T2DM due to its high mortality rate, ranging from 5 to 20% [7]. The mortality of HHS in the elderly or unconscious patients could be even higher [8, 9]. Despite the overall improvement in treating hyperglycemic crisis (including HHS and DKA) [10], the mortality was still high, especially with HHS [11]. The prognostic factors were variable in different populations in previous studies, such as age, the presence of cardiovascular comorbidities, sodium and bicarbonates [7, 8]. So far, data on the exact predisposing risk factors are still lacking in Chinese patients. Previous studies have reported that some patients with HEs could have combined features of HHS and DKA [12, 13]. However, the characteristics and prognosis of patients with combined DKA-HHS in China are still largely unclear. Hence forth, our study sought to identify potential predictors of mortality for Chinese patients with HHS in order to effectively improve their in-hospital management and decrease associated mortality.

Here, we analyzed clinical characteristics and treatment outcomes of T2DM patients hospitalized for isolated or combined HEs in a tertiary teaching hospital in Shanghai, China, and evaluated the predisposing factors implicated in the severity and mortality of patients.

## Methods

### Patients

T2DM patients admitted to Shanghai Tongji Hospital because of DKA and HHS from January 2010 to December 2015 were included in the present study. Informed consent was obtained from all participants. This study was conducted in accordance with the Declaration of Helsinki and approved by the local ethics committee (Tongji Hospital of Tongji University).

Criteria of DKA: Those who had or no history of T2DM, hyperglycemia (random blood glucose, RBG usually > 16.7 mmol/L), positive or strongly positive urine or blood ketone, and arterial PH < 7.30 or blood bicarbonate < 18 mmol/L. According to an ADA consensus on acute complications of diabetes in 2009 [2], DKA is classified as mild, moderate, and severe based on the severity of metabolic acidosis (blood PH, bicarbonate, and ketones) and the presence of altered mental status: Mild DKA: a pH of 7.25–7.29 and (or) a bicarbonate of 15–17.9 mmol/L and anion gap(AG) > 10; Moderate DKA: a pH of 7.00–7.24 and (or) a bicarbonate of 10–14.9 mmol/L and AG > 12; Severe DKA: DKA complicated with conscious disturbance (DKA coma), or arterial PH < 7.00 and (or) blood bicarbonate < 10 mmol/L and AG > 12 without DKA coma.

Criteria of HHS: Hyperglycemia (RBG > 33.3 mmol/L), venous PH > 7.25 or arterial PH > 7.30, blood bicarbonate > 18 mmol/L, AG < 12, strongly positive urine glucose, negative or weakly positive urine ketone, normal or slightly higher blood ketone, effective plasma osmolality > 320 mmol/L, and complicated with conscious disturbance, delirium or epileptiform convulsion [2].

Criteria of DKA plus HHS: RBG > 33.3 mmol/L, positive or strongly positive urine or blood ketone, arterial PH < 7.30 or blood bicarbonate < 18 mmol/L, effective plasma osmolality > 320 mmol/L, and complicated with conscious disturbance, delirium or epileptiform convulsion [2, 14].

Treatments: Physicians in charge of the patients followed China guideline for type 2 diabetes. All the patients were given continuous intravenous low-dose insulin infusion firstly: 0.1 units/kg/h as an initial rate of intravenous insulin infusion, then adjusting according to the blood glucose concentration and rate of blood glucose decline. When the patient was much improved clinically and was able to eat adequately, subcutaneous insulin therapy such as continuous subcutaneous insulin infusion (CSII) or multiple daily injections (MDI) was then introduced. American Medtronic 508 Portable pulse insulin pumps with insulin aspart were installed in CSII groups, while insulin aspart and insulin glargine were used in MDI group.

Medical history of patients included gender, age, duration of diabetes, duration of hospital stay, precipitants, chronic diabetic complications (peripheral neuropathy, retinopathy, nephropathy, peripheral vascular disease), co-morbidities (hypertension, hyperlipidemia, hyperuricemia, liver and kidney disease, coronary heart disease, cerebrovascular disease), history of smoking and drinking, and family medical history.

Clinical features: On-admission clinical presentations (temperature, pulse rate, respiration rate, blood pressure, consciousness), laboratory parameters included blood glucose (BG), effective plasma osmolality (EPO), serum potassium (K^+^), serum sodium (Na^+^), hemoglobin (Hb), white blood cell count (WBC), red blood cell count (RBC), C-reaction protein (CRP), procalcitonin (PCT), myohemoglobin (MYO), creatine kinase MB (CK-MB), troponin I (Tn-I), Natriuretic peptide (BNP), urea nitrogen (BUN), creatinine (Cr), uric acid (UA), serum calcium (Ca), serum phosphorus (P), osteocalcin, glycosylated hemoglobin (HbA1c), glycated albumin (GA-L), total protein, albumin, alanine transaminase (ALT), aspartate amino transferase (AST), triglyceride (TG), total cholesterol (TC), high-density lipoprotein cholesterol (HDL-c), low density lipoprotein cholesterol (LDL-c), arterial PH, blood standard bicarbonate (SB), fasting C-peptide, fasting insulin, D-Dimer, fibrinogen, prothrombin time (PT), activated partial thromboplastin time (APTT). Effective plasma osmolality (EPO) (mOsm/L) =2 × [Na^+^ + K^+^](mmol/L) + BG (mmol/L). Other examinations included: lower extremity vascular ultrasound and abdomen ultrasound.

The main outcome of this study was mortality due to DKA and HHS, and secondary outcomes were glycemic control including FBG before discharge and hypoglycemic events, length of hospital stay, and expenditure during hospital. Hypoglycemia was defined as capillary glucose ≤3.9 mmol/L or symptoms of hypoglycemia could be resolved with administration of oral carbohydrates if glucose was not measured. Severe hypoglycemia was defined as a capillary glucose <2.8 mmol/l associated with confusion, loss of consciousness, or seizures [15].

### Statistical analysis

All results were performed using the statistical package for social sciences for windows (SPSS, version 18.0, Chicago, Illinois, USA). Continuous variables were analyzed with the Kolmogorov-Smirnov test to determine whether they followed a normal distribution. All continuous variables conforming to normal distribution were presented as mean ± standard deviation (SD), whereas data that did not have a normal distribution, were illustrated as medians (interquartile ranges). All categorical variables were presented as proportions. Continuous variables that didn’t follow a normal distribution were log transformed to approximate normal distribution for further analysis. Comparisons of the means between two groups were performed with independent samples T-test. Comparisons of the means among three or more groups were performed with one-way ANOVA and the least significant differences (LSD) post hoc multiple comparison test. Comparisons of the proportions were analyzed using the chi-square test. Spearman’s correlations and logistic regression analysis were performed to explore the link between risk factors and mortality in patients with HHS. A receiver operating characteristic (ROC) analysis was conducted to determine the capacity of the clinical and biochemistry markers in predicting all-cause mortality in patients with HHS. Tests were two-sided and differences were considered statistically significant if *P*-value < 0.05.

## Results

A total of 158 diabetic patients who were admitted to Shanghai Tongji Hospital because of hyperglycemic crises were recruited in this study. The average age was 64.8 ± 17.7 years (range: 20–95 years). 44.9% (71/158) were male and 55.1% (87/158) were female. Overall, 41.1% (65/158) of the patients were DKA, 46.8% (74/158) were HHS, while 12.0% (19/158) were DKA plus HHS (DKA-HHS). 17.7% (28/158) of the patients were newly diagnosed diabetes on admission and the remaining were known diabetic patients on follow up at Shanghai Tongji Hospital. Most of patients (50.6%) had diabetes for 1 to 10 years, 24.1% (38/158) patients had diabetes for more than 10 years, while only 7.6% (12/158) of patients had diabetes for less than 1 year. The majority, 57.1% (16/28), of the newly diagnosed T2DM patients developed HHS, 39.3% (11/28) with DKA, and 3.6% (1/28) with DKA-HHS. The RBG was 28.6 ± 7.8 mmol/L, and HbA1C was 11.1 ± 2.8% on admission. The top three precipitating factors were infections (111/158, 70.3%), including pulmonary infections, urinary tract infections, decubital ulcers and multiple infections, newly diagnosed diabetes (28/158, 17.7%), and non-compliance to medications (9/158, 5.7%).

Admission characteristics of the study population are shown in Table 1. Patients with HHS were significantly older than those with DKA (75.4 ± 11.6 vs 53.4 ± 16.5 years, *P* < 0.001). The mean length of hospital stay was 14.7 days, 11.5 days for DKA patients, 18.3 days for HHS, and 11.6 days for DKA-HHS. The total expenses of HHS patients during hospital stay were more expensive than those of DKA group. The proportion of patients with newly diagnosed diabetes was higher in the HHS group, and the majority of patients with infections was in the HHS group as well. As expected, patients with HHS had higher BG, EPO, BUN, Cr, Na^+^, arterial PH and SB than DKA group. The overall in-hospital mortality due to HEs among all subjects was 17 (10.8%). The highest mortality rate was observed in HHS group, which was (16/74) 21.6%, whereas no death was recorded in patients with DKA. Mortality among previously known diabetic patients (14, 10.8%) was similar to newly diagnosed ones (3, 10.7%). There was no significant difference in the number of deaths between males and females (8 of male and 9 of female).

**Table 1:** Admission characteristics of diabetic patients by type of HEs(*X ± S*)

Variables	DKA	HHS	DKA-HHS
Number of patients	65	74	19
Age (years)	53.4 ± 16.5	75.4 ± 11.6 ^aa^	61.9 ± 16.9 ^c^
Gender (male: female)	38:27	26:48	7:12
History of DM (years)	7.01 ± 6.42	8.0 (0.5–10.0)	10.39 ± 9.52
Hospital stay (days)	11.49 ± 5.10	18.32 ± 14.50 ^aa^	11.58 ± 6.57 ^c^
Expenses (Yuan)	8955.3 (6583.5−12,838.1)	14,815.3 (7387.8−20,449.7) ^aa^	10,827.5 ± 5280.5 ^cc^
Mortality (%)	0	21.6	5.3
RBG (mmol/L)	20.64 ± 6.02	34.27 ± 1.78 ^aa^	33.98 ± 0.72^bb^
HbA1c(%)	11.60 ± 2.58	10.68 ± 2.77	10.86 ± 3.18
Fasting C-peptide (ng/ml)	0.35 (0.12–0.80)	0.95 ± 0.81 ^a^	0.28 (0.09–0.37) ^c^
Arterial PH	7.21 ± 0.10	7.40 ± 0.06 ^aa^	7.15 ± 0.11 ^cc^
SB (mmol/L)	12.82 ± 4.69	23.50 ± 4.55 ^aa^	12.58 ± 7.74 ^cc^
EPO (mOsm/L)	308.82 ± 14.21	383.74 ± 22.12 ^aa^	348.37 ± 31.94 ^bb, cc^
Na^+^(mmol/L)	135.92 ± 5.67	160.19 ± 8.28 ^aa^	146.05 ± 12.96 ^bb,cc^
BUN (mmol/L)	7.98 ± 5.22	21.01 ± 11.60 ^aa^	14.17 ± 8.38 ^b,c^
Cr (umol/L)	79.0 (60.0–107.50)	151.50 (82.50−218.0) ^aa^	163.16 ± 66.79 ^bb^

Note: DKA vs HHS,^a^ *P* < 0.05, ^aa^ *P* < 0.01

DKA vs DKA-HHS, ^b^ *P* < 0.05, ^bb^ *P* < 0.01

HHS vs DKA-HHS, ^c^ *P* < 0.05, ^cc^ *P* < 0.01

Mild, moderate, and severe DKA accounted for 38.5% (25/65), 32.3% (21/65), and 29.2% (19/65) of the patients, respectively. Table 2 shows the clinical features of mild, moderate and severe groups of DKA. Patients with severe DKA were significantly younger than those with mild DKA (46.3 ± 13.0 vs 59.0 ± 18.4 years, *P* = 0.011). The majority of mild DKA patients were female, while most of patients in moderate and severe groups were male. The mean expenditure was 9958.0, 11,085.9, and 12,336.4 yuan, for those with mild, moderate, and severe DKA, corresponding to a mean hospital stay of 9.89, 11.14 and 13 days, respectively. Infections were the most common precipitating factor, underlying 58.5% (38) of all those with DKA, accounting for 52, 61.9, and 63.2% in those with mild, moderate, and severe DKA, respectively. The proportion of patients with newly diagnosed diabetes was similar in mild DKA and severe DKA group (20.0% vs 21.1%).

**Table 2:** Clinical features of mild, moderate and severe groups of DKA on admission

Variables	Mild group	Moderate group	Severe group
Number of patients	25	21	19
Age (years)	59.0 ± 18.4	53.2 ± 14.9	46.3 ± 13.0 ^b^
Gender (male: female)	11:14	14:7	13:6
History of DM (years)	7.62 ± 6.93	8.32 ± 5.93	4.77 ± 5.99
Hospital stay (days)	9.89 ± 5.86	11.14 ± 4.86	13.00 ± 4.40 ^b^
Expenses (Yuan)	9958.0 ± 7267.5	11,085.9 ± 7952.6	12,336.4 ± 8080.6
RBG (mmol/L)	20.01 ± 5.32	20.45 ± 5.81	21.67 ± 7.20
HbA1c(%)	11.63 ± 2.66	11.47 ± 2.65	11.69 ± 2.52
Fasting C-peptide (ng/mL)	1.00 ± 0.89	0.39 ± 0.35 ^a^	0.32 ± 0.31^bb^
Arterial PH	7.30 ± 0.02	7.21 ± 0.05 ^aa^	7.11 ± 0.09 ^bb,cc^
SB (mmol/L)	17.46 ± 2.23	12.37 ± 1.47 ^aa^	7.20 ± 2.15 ^bb,cc^
EPO (mOsm/L)	307.20 ± 14.21	310.66 ± 16.65	319.03 ± 11.72
Na (mmol/L)	135.84 ± 4.66	136.85 ± 6.08	135.05 ± 6.54
BUN (mmol/L)	7.65 ± 6.15	8.29 ± 4.86	8.09 ± 4.44
Cr (umol/L)	77.00 ± 25.05	102.52 ± 76.06	123.74 ± 83.72 ^b^
CRP (mg/L)	10.50 (7.0–28.50)	67.19 ± 52.06	91.60 ± 64.79 ^bb^

Note: mild group vs moderate group, ^a^ *P* < 0.05, ^aa^ *P* < 0.01

mild group vs severe group, ^b^ *P* < 0.05, ^bb^ *P* < 0.01

moderate group vs severe group, ^cc^ *P* < 0.01

As expected, patients with severe DKA had lower arterial PH and SB, and longer hospital stay than mild group. In addition, compared with mild patients, Cr and CRP were significantly higher in patients with severe DKA, whereas age and fasting C peptide were remarkably lower. Spearman rank correlation analysis revealed CRP was positively correlated with the severity of DKA, whereas age and fasting C peptide were inversely correlated with the severity of DKA (*P* < 0.05).

Our data also showed that total mortality of HEs was 10.8%, and total mortality of HHS was 21.6%. The highest mortality rate was observed in age groups > 80 years (8, 26.7%), whereas the lowest mortality rate occurred in age groups < 70 years (3, 13.6%). The mortality recorded in patients with age 70–80 was 22.7% (5/22). Mortality rate in males was higher than in females (26.9 vs. 18.8%), however, it was not statistically significant. The highest mortality (4, 25.0%) was recorded in patients with > 10 years duration of diabetes and antidiabetic treatment, whereas the lowest mortality rate occurred in patients with < 1 year duration of diabetes (3, 15.8%). Among the 16 non-survivors of HHS, 10 (62.5%) died in hospital within 7 days after admission. Mostly were induced by infections, among which 71.4% (10/14) had pulmonary infections, 14.3% (2/14) had urinary tract infections, 14.3% (2/14) had decubital ulcers and 28.6% (4/14) had multiple infections.

When compared with survivors, BUN, Cr and EPO were significantly increased in non-survivors (*P* < 0.05) (Table 3). Spearman correlation analysis was shown in Table 4, which suggested that mortality was positively correlated to EPO, renal function indicators (BUN, Cr) and hepatic enzymes (ALT, AST). To further investigate the associations between mortality and those five variables, unadjusted and multivariate adjusted logistic regression analyses were used. The mortality was significantly associated with increased levels of BUN (OR: 1.095, 95% CI: 1.007–1.191, *p* = 0.035). The association persisted after further adjustment for age and gender (OR: 1.103, 95% CI: 1.010–1.205, *p* = 0.03).

**Table 3:** Clinical data of patients with HHS on admission

Variables	Survivors	Non-survivors
Number of patients	58	16
Age (year)	74.7 ± 12.3	78.3 ± 8.2
Gender (male: female)	19:39	7:9
History of DM (years)	7.47 ± 6.73	3 (2–10)
Hospital stay (days)	19.67 ± 12.90	5.5 (3–19.75)
Expenses (Yuan)	16,988.2 ± 10,927.4	8680.5 (2974.6–23,185.4)
RBG (mmol/L)	34.44 ± 1.96	33.66 ± 0.57
HbA1c(%)	10.58 ± 2.64	11.46 ± 3.74
Fasting C-peptide (ng/mL)	0.95 ± 0.68	0.31 (0.10–2.27)
EPO (mOsm/L)	380.76 ± 20.93	394.58 ± 23.61^*^
Na + (mmol/L)	169.88 ± 8.34	161.31 ± 8.19
BUN (mmol/L)	18.61 ± 10.05	29.70 ± 12.98^**^
Cr (umol/L)	151.86 ± 86.25	194.5 (159–232.75)^*^
ALT(U/L)	21.5 (13–32.25)	36.5 (21.25–86.25)
AST(U/L)	23 (16–37.5)	35.0 (23.0–145.0)
Percentage of CSII therapy (%)	50.0 (29/58)	18.8 (3/16)^*^

* *P*-value < 0.05, ** *P*-value < 0.01

**Table 4:** Spearman’s correlations between variables and mortality in patients with HHS

Parameter		Age	Gender	History of DM	RBG	EPO	BUN	Cr	ALT	AST	CSII therapy
Mortality	*R*	0.108	− 0.095	0.002	−0.214	− 0.256*	0.353**	0.233*	0.335**	0.319**	−0.26*
	*P*	0.358	0.422	0.989	0.067	0.028	0.002	0.046	0.004	0.006	0.025

* *P*-value < 0.05, ** *P*-value < 0.01

**Fig. 1:**
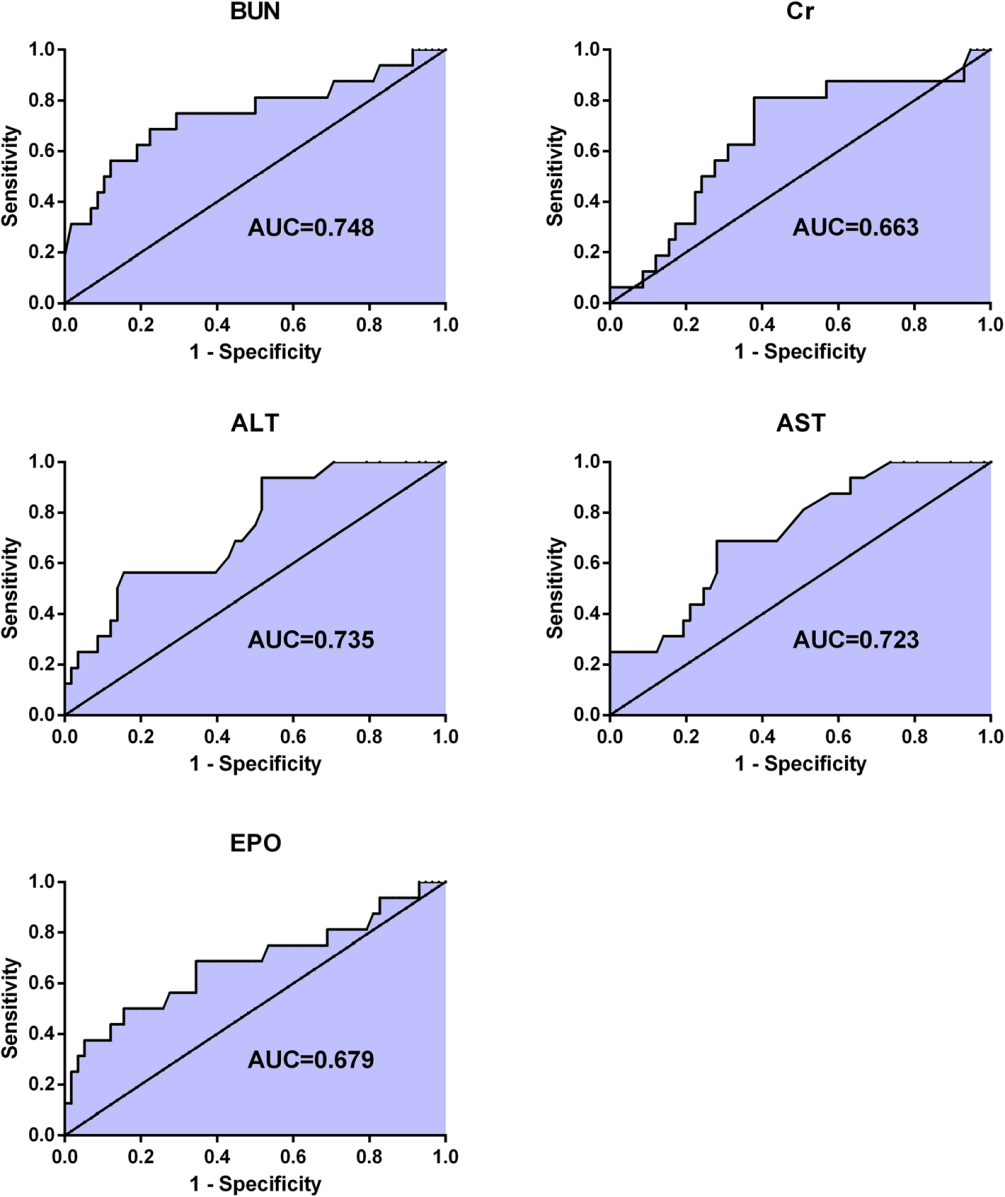
ROC curves showing the diagnostic performance of six variables in predicting all-cause mortality in patients with HHS. Larger test result indicates more positive test. CI, confidence interval. The area under the ROC curve (AUC) = 0.748 for BUN, *P* = 0.003, 95%CI 0.593–0.903. AUC = 0.679 for EPO, *P* = 0.029, 95%CI 0.512–0.846; AUC = 0.663 for Cr, *P* = 0.047, 95%CI 0.513–0.814; AUC = 0.735 for ALT, *P* = 0.004, 95%CI 0.604–0.866; AUC = 0.723 for AST, *P* = 0.007, 95%CI 0.591–0.854

Furthermore, ROC curves were constructed to analyze the sensitivity and specificity of those candidate variables individually for estimation of death in HHS patients. The areas under the ROC curves (AUCs) of the five variables were displayed in Fig. 1. As demonstrated by the ROC curves, BUN displayed the best sensitivity and specificity in discriminating the non-survivors from the rest of patients in HHS group (AUC = 0.748, *P* = 0.003, 95% confidence interval 0.593–0.903) among the five candidate variables for detection of non-survivors. The ROC analysis revealed that BUN levels > 24.75 mmol/L had good capacity to differentiate the non-survivors from survivors in HHS group. This cut-off point for BUN level had a sensitivity of 68.8%, and a specificity of 77.6% for predicting mortality in HHS patients. 68.8% (11/16) non-survivors of HHS had a BUN > 24.75 mmol/L, while only 22.4% (13/58) survivors of HHS had a BUN > 24.75 mmol/L.

Among the 74 HHS patients, all patients were received continuous intravenous low-dose insulin infusion in the early stage of treatment according to China guideline for type 2 diabetes. When the patient was much improved clinically, subcutaneous insulin therapy was performed. We found 32 (43.2%) was received CSII therapy, whereas 42(56.8%) without CSII therapy, including 34 (45.9%) with multiple daily injection (MDI) therapy, and 8 (10.8%) was only given continuous intravenous insulin infusion. The mortality rate in patients using CSII was significantly lower than those without CSII therapy (9.4% vs 31%, *P* < 0.05). There was a trend toward a lower mortality in the CSII group than MDI group (9.4% vs 14.7%), however, no significant difference between CSII and MDI group was found. Spearman correlation analysis suggested that death was inversely associated with CSII therapy. In addition, unadjusted and multivariate adjusted logistic regression analyses indicated CSII was a protective factor (*P* < 0.05). The mortality rate was inversely associated with CSII therapy (OR:0.231, 95% CI: 0.059–0.896, *p* = 0.034). The association persisted after further adjustment for age and gender (OR: 0.246, 95% CI: 0.062–0.971, *p* = 0.045).

We also compared the efficacy and safety of CSII and MDI in survivors with HHS before discharge (Table 5). We found CSII gained better glycemic control over MDI in survivors with HHS. In our study, average FBG levels/values before discharge in CSII group was significantly lower than those in MDI group. There was no significant difference in the total number of hypoglycemic events or the number of subjects experiencing hypoglycemia between the CSII and MDI groups. Nevertheless, there was a higher percentage of severe hypoglycemia in the MDI group than CSII. Furthermore, no significant difference in the length of hospital stay and expenses between CSII and MDI group was found.

**Table 5:** Clinical data of survivors with HHS before discharge

Variables	MDI	CSII
Number of patients	29	29
Age (year)	74.8 ± 12.5	74.5 ± 12.3
Gender (male: female)	11:18	8:21
Hospital stay (days)	19.83 ± 14.68	19.52 ± 11.11
Expenses (Yuan)	15,836.9 ± 6302.6	18,139.4 ± 14,163.8
FBG (mmol/L)	10.68 ± 3.47	8.54 ± 2.77*
EPO (mOsm/L)	298.70 ± 10.87	300.87 ± 8.59
Na^+^(mmol/L)	137.76 ± 3.50	139.62 ± 3.37
BUN (mmol/L)	5.99 ± 4.49	5.82 ± 3.12
Cr (umol/L)	75.75 ± 34.10	73.15 ± 30.57
Total number of times that blood glucose monitoring was performed during MDI or CSII therapy	1523	1756
Total number of hypoglycemic events during MDI or CSII therapy	15	16
Percentage of Severe hypoglycemic events (%)	26.7 (4/15)	0 (0/16)*
Number of subjects experiencing hypoglycemia	7	5

*P-value < 0.05

## Discussion

Our study found that HHS was the most common acute complication in inpatients with T2DM, and newly diagnosed diabetes accounted for the second common precipitating factor (17.7%) of HEs. Previous studies reported that DKA and HHS were the first manifestations in nearly 20% newly diagnosed diabetic patients [16–18], which was similar to our result. Hence, there is a need for more public awareness of diabetes and strengthening diabetes screening network in China, especially for those with risk factors of diabetes.

The mortality rate of DKA varies greatly in previous studies, usually ranging from 2 to 5% [13, 19], but was reported as being more than 5% in the elderly and those with associated life-threatening conditions [2, 20]. In our study, none of DKA patients died, this mortality rate of DKA was close to some developed countries such as USA and UK [21, 22], but much lower than in many developing countries [23, 24].

Patients with severe DKA had longer duration of hospital stay compared with patients with mild DKA, which was similar to other studies [25]. The most common precipitating factor of DKA in our study was infection, which was closely approximated other developing countries [13, 20, 26, 27]. In contrast, change of regimen and newly detected diabetes were the most common precipitating factors in USA and other developed countries [26, 28–30]. Thus, our study showed unique characteristics of DKA in China, which were quite different from other countries.

HHS is a serious lethal state of hyperglycemia with different degrees of conscious disturbances, ranging from lethargy to coma, which differs from DKA. HHS is the most dangerous complication of T2DM due to its high mortality rate [31], which was reported to be between 10 and 40% [7–9, 32, 33]. In our study, total mortality of HHS was much higher than DKA, which was 21.6%. Our study also found HHS occurs more commonly in elderly female patients, with the majority of patients having infections as the leading precipitating factor. As reported in previous studies, advanced age, male gender, high glucose, high leukocyte count, low bicarbonates, and altered mental status were associated with increased mortality of HHS [8, 34]. In our study, we revealed a novel prognostic factor and a new protective factor for HHS patients. We found elevated BUN might serve as an independent predictor of mortality in patients with HHS. Our study also compared the impact of CSII and MDI on treatment outcomes in patients with HHS. CSII was effective in the intensive control of hyperglycemia in patients with DM. Studies comparing CSII and MDI in patients with T1DM and T2DM have either found comparable outcomes or favored CSII [15, 35–37]. However, the efficacy and safety of CSII and MDI have not been compared in T2DM patients with HHS. Our study confirmed that CSII offered significant benefits over MDI for individuals with HHS, with decrease in mortality as well as improvements in glycemic control. We found that the mortality rate of CSII group in HHS patients was much lower than MDI group, and FBG before discharge in survivors was significantly decreased in CSII group compared with MDI group. There was also a lower number of severe hypoglycemic events in CSII group than MDI.

Meanwhile, our research has certain limitations. Firstly, the sample size was small, and from a single medical center. Secondly, this was a retrospective study, without long-term outcomes. Thus, future multicenter and follow-up studies are needed to re-assess our findings in a larger cohort of patients.

In summary, our study revealed the high mortality of HHS in China, whereas the mortality rate of DKA was very low/ negligible. Elevated CRP and decreased fasting C-peptide on admission might serve as risk factors of severe DKA. Elevated BUN might be an independent predictor of mortality in patients with HHS, whereas CSII could be a protective factor against death. Our study could be valuable in predicting mortality of HEs, allowing interventions to be targeted most effectively to reduce associated mortality.

## Data Availability

The datasets used and analysed during the current study are available from the corresponding author on reasonable request.

## References

[1] Roglic G, Unwin N, Bennett PH, Mathers C, Tuomilehto J, Nag S (2005). The burden of mortality attributable to diabetes: realistic estimates for the year 2000. Diabetes Care.

[2] Kitabchi AE, Umpierrez GE, Miles JM, Fisher JN (2009). Hyperglycemic crises in adult patients with diabetes. Diabetes Care.

[3] Wang ZH, Kihl-Selstam E, Eriksson JW (2008). Ketoacidosis occurs in both type 1 and type 2 diabetes--a population-based study from northern Sweden. Diabet Med.

[4] Ezeani I, Eregie A, Ogedengbe O (2013). Treatment outcome and prognostic indices in patients with hyperglycemic emergencies. Diabetes Metab Syndr Obes.

[5] Miyake Y (2014). Management of Hyperglycemic Crises and Severe Hypoglycemia in the Emergency Department. Brain Nerve.

[6] Ogbera AO, Awobusuyi J, Unachukwu C, Fasanmade O (2009). Clinical Features, Predictive Factors and Outcome of Hyperglycaemic Emergencies in a Developing Country. BMC Endocr Disord.

[7] Fadini GP, de Kreutzenberg SV, Rigato M, Brocco S, Marchesan M, Tiengo A (2011). Characteristics and outcomes of the hyperglycemic hyperosmolar non-Ketotic syndrome in a cohort of 51 consecutive cases at a single center. Diabetes Res Clin Pract.

[8] Chung ST, Perue GG, Johnson A, Younger N, Hoo CS, Pascoe RW (2006). Predictors of Hyperglycaemic crises and their associated mortality in Jamaica. Diabetes Res Clin Pract.

[9] Chu CH, Lee JK, Lam HC, Lu CC (2001). Prognostic factors of hyperglycemic hyperosmolar nonketotic state. Chang Gung Med J.

[10] Wei NJ, Wexler DJ, Nathan DM, Grant RW (2013). Intensification of diabetes medication and risk for 30-day readmission. Diabet Med.

[11] Liu C-C, Chen K-R, Chen H-F, Huang H-L, Ko M-C, Li C-Y (2010). Trends in hospitalization for diabetic ketoacidosis in diabetic patients in Taiwan: analysis of national claims data. J Formos Med Assoc.

[12] Pasquel FJ, Tsegka K, Wang H, Cardona S, Galindo RJ, Fayfman M (2020). Clinical outcomes in patients with isolated or combined diabetic ketoacidosis and hyperosmolar hyperglycemic state: a retrospective, hospital-based cohort study. Diabetes Care.

[13] Kitabchi AE, Umpierrez GE, Murphy MB, Barrett EJ, Kreisberg RA, Malone JI (2001). Management of Hyperglycemic Crises in patients with diabetes. Diabetes Care.

[14] Wild S, Roglic G, Green A, Sicree R, King H (2004). Global prevalence of diabetes: estimates for the year 2000 and projections for 2030. Diabetes Care.

[15] Herman WH, Ilag LL, Johnson SL, Martin CL, Sinding J, Al Harthi A (2005). A clinical trial of continuous subcutaneous insulin infusion versus multiple daily injections in older adults with type 2 diabetes. Diabetes Care.

[16] Desse TA, Eshetie TC, Gudina EK. Predictors and treatment outcome of hyperglycemic emergencies at Jimma University Specialized Hospital, southwest Ethiopia. BMC Res Notes. 2015;8. 10.1186/s13104-015-1495-z. 10.1186/s13104-015-1495-z PMC4601134 26455633

[17] Umpierrez GE, Kelly JP, Navarrete JE, Casals MM, Kitabchi AE. Hyperglycemic crises in urban blacks. Arch Intern Med. 1997;157(6). 10.1001/archinte.1997.00440270117011. 9080921

[18] Pasquel FJ, Umpierrez GE (2014). Hyperosmolar hyperglycemic state: a historic review of the clinical presentation, diagnosis, and treatment. Diabetes Care.

[19] Graves EJ, Kozak LJ (1998). Detailed Diagnoses and Procedures, National Hospital Discharge Survey, 1996. Vital Health Stat 13.

[20] Seth P, Kaur H, Kaur M (2015). Clinical profile of diabetic ketoacidosis: a prospective study in a tertiary care hospital. J Clin Diagn Res.

[21] Kitabchi AE, Nyenwe EA (2006). Hyperglycemic crises in diabetes mellitus: diabetic ketoacidosis and hyperglycemic hyperosmolar state. Endocrinol Metab Clin N Am.

[22] Gibb FW, Teoh WL, Graham J, Lockman KA (2016). Risk of death following admission to a UK Hospital with diabetic ketoacidosis. Diabetologia..

[23] Mbugua PK, Otieno CF, Kayima JK, Amayo AA, McLigeyo SO (2005). Diabetic ketoacidosis: clinical presentation and precipitating factors at Kenyatta National Hospital. Nairobi East Afr Med J.

[24] Poovazhagi V (2014). Risk Factors for Mortality in Children With Diabetic Keto Acidosis From Developing Countries. World J Diabetes.

[25] Basetty S, Kumar GSY, Shalini M, Angeline RP, David KV, Abraham S (2017). Management of Diabetic Ketosis and Ketoacidosis with Intramuscular Regular Insulin in a low-resource family medicine setting. J Family Med Prim Care.

[26] Umpierrez G, Korytkowski M (2016). Diabetic emergencies - ketoacidosis, Hyperglycaemic hyperosmolar state and Hypoglycaemia. Nat Rev Endocrinol.

[27] Jabbar A, Farooqui K, Habib A, Islam N, Haque N, Akhter J (2004). Clinical characteristics and outcomes of diabetic ketoacidosis in Pakistani adults with type 2 diabetes mellitus. Diabet Med.

[28] Freire AX, Umpierrez GE, Afessa B, Latif KA, Bridges L, Kitabchi AE (2002). Predictors of intensive care unit and hospital length of stay in diabetic ketoacidosis. J Crit Care.

[29] Azevedo LCP, Choi H, Simmonds K, Davidow J, Bagshaw SM (2014). Incidence and long-term outcomes of critically ill adult patients with moderate-to-severe diabetic ketoacidosis: retrospective matched cohort study. J Crit Care.

[30] Barski L, Nevzorov R, Rabaev E, Jotkowitz A, Harman-Boehm I, Zektser M (2012). Diabetic ketoacidosis: clinical characteristics, precipitating factors and outcomes of care. Isr Med Assoc J.

[31] Trence DL, Hirsch IB (2001). Hyperglycemic crises in diabetes mellitus type 2. Endocrinol Metab Clin N Am.

[32] Kitabchi AE, Umpierrez GE, Murphy MB, Barrett EJ, Kreisberg RA, Malone JI (2004). Hyperglycemic Crises in Diabetes. Diabetes Care.

[33] Kitabchi AE, Umpierrez GE, Murphy MB, Kreisberg RA (2006). Hyperglycemic crises in adult patients with diabetes: a consensus statement from the American Diabetes Association. Diabetes Care.

[34] Kruljac I, Ćaćić M, Ćaćić P, Biloš LSK, Kust D, Perić B (2018). The impact of Hyperosmolarity on long-term outcome in patients presenting with severe hyperglycemic crisis: a population based study. Exp Clin Endocrinol Diabetes.

[35] Doyle EA, Weinzimer SA, Steffen AT, Ahern JAH, Vincent M, Tamborlane WV (2004). A randomized, prospective trial comparing the efficacy of continuous subcutaneous insulin infusion with multiple daily injections using insulin Glargine. Diabetes Care.

[36] J Hans DeVries, Frank J Snoek, Piet J Kostense, Nathalie Masurel, Robert J Heine, Dutch Insulin Pump Study Group. A Randomized Trial of Continuous Subcutaneous Insulin Infusion and Intensive Injection Therapy in Type 1 Diabetes for Patients With Long-Standing Poor Glycemic Control. Diabetes Care. 2002 ;25(11):2074–2080. 10.2337/diacare.25.11.2074. 10.2337/diacare.25.11.2074 12401759

[37] Raskin P, Bode BW, Marks JB, Hirsch IB, Weinstein RL, McGill JB (2003). Continuous subcutaneous insulin infusion and multiple daily injection therapy are equally effective in type 2 diabetes: a randomized, parallel-group, 24-week study. Diabetes Care.

